# Kinesin 5B (KIF5B) Is Required for Progression through Female Meiosis and Proper Chromosomal Segregation in Mitotic Cells

**DOI:** 10.1371/journal.pone.0058585

**Published:** 2013-04-01

**Authors:** Dawit Kidane, Denny Sakkas, Timothy Nottoli, James McGrath, Joann B. Sweasy

**Affiliations:** 1 Departments of Therapeutic Radiology and Genetics and The Yale Comprehensive Cancer Center, New Haven, Connecticut, United States of America; 2 Yale Animal Genomics Services, Comparative Medicine Faculty, Yale School of Medicine, New Haven, Connecticut, United States of America; 3 Departments of Obstetrics, Gynecology and Reproductive Sciences, Yale School of Medicine, New Haven, Connecticut, United States of America; University of Connecticut, United States of America

## Abstract

The fidelity of chromosomal segregation during cell division is important to maintain chromosomal stability in order to prevent cancer and birth defects. Although several spindle-associated molecular motors have been shown to be essential for cell division, only a few chromosome arm-associated motors have been described. Here, we investigated the role of Kinesin 5b *(Kif5b)* during female mouse meiotic cell development and mitotic cell division. RNA interference (RNAi)-mediated silencing of *Kif5b* in mouse oocytes induced significant delay in germinal vesicle breakdown (GVBD) and failure in extrusion of the first polar body (PBE). In mitotic cells, knockdown of *Kif5b* leads to centrosome amplification and a chromosomal segregation defect. These data suggest that KIF5B is critical in suppressing chromosomal instability at the early stages of female meiotic cell development and mitotic cell division.

## Introduction

Spatial and temporal control of cell division is essential to ensure the equal segregation of genetic material between daughter cells. Chromosome segregation in mitosis and meiosis is directed by kinetochores, which regulate the attachment and movement of chromosomes on the spindle and ensure the fidelity of segregation [1], [2]. Defective kinetochore function leads to elevated rates of chromosome loss or gain. In meiosis, disjunction of maternal from paternal centromeres depends on the attachment of sister kinetochores to microtubules emanating from the same pole. Remodeling of chromosomes during oocyte meiosis begins when homologues initially pair and condense via actions of the synaptonemal complex proteins (SC) during initiation of prophase I, followed by homologous recombination and crossing-over events [3]. Chromatin subsequently decondenses as oocytes enter a phase of quiescence prior to completing prophase I. Meiotic spindles in mammalian oocytes lack centrioles, which are present only up to the pachytene stage during oogenesis [4]. A bipolar meiotic spindle forms, consisting of polymerized microtubules, and attaches to homologues at their centromeres. Subsequently, physical contact between homologous pairs at chiasmata counteracts forces pulling apart homologues, resulting in alignment of chromosomes along the metaphase plate, signaling completion of metaphase I (MI). Forces moving toward and away from the pole balance each other during metaphase congression and are responsible for chromosome motility toward the poles [5].

The dynamic nature of the spindle apparatus is believed to be maintained both by the instability of microtubules as well as several force-producing microtubule motors [6]. Polar ejection forces may be generated either by microtubulin or by the plus-end-directed motor Kinesin, which associates with chromosome arms [7], [8]. The meiotic spindle then facilitates separation and segregation as homologues are pulled toward opposite spindle poles at the beginning of the Anaphase stage of Meiosis I. However the first meiotic division is unique in that it is reductional: homologous chromosomes that just underwent meiotic recombination are segregated, while the cohesion between sister chromatids is maintained. Oocytes progress through telophase, resulting in the formation of a cleavage furrow between the two daughter cells leading to disproportionate cytokinesis and extrusion of the first polar body and signaling completion of meiosis I [9], [10].

Kinesin proteins, in addition to their transport roles, influence microtubule dynamics, kinetochore microtubule attachment, and centrosome separation [11]. In addition, kinesins are involved in a wide array of cellular functions by coupling ATP hydrolysis to the regulated and targeted movement of specific intracellular cargo along microtubule filaments [12], [13]. Recently, transcription-dependent fusions of KIF5B-RET have been found to lead to aberrant activation of RET kinase in what could be considered to be a new driver mutation of lung adenocarcinoma [14]. However, the biological significance of the interaction between kinesin proteins and centromeric chromatin in building a functional kinetochore remains unclear. KIF5B protein is one of the molecular motors that engages in the transport of RNA in neurons and interacts with Glutamate Receptor Interacting Protein-1 (GRIP1), a scaffold protein composed of seven PDZ (Postsynaptic synaptic density-95/Discs large/Zona occludens-1) domains [15], [16]. Targeted disruption of KIF5B in mice leads to embryonic lethality [17].

In this study, we characterize the subcellular localization and function of KIF5B in directing female meiotic cell development and mitotic cell division. Downregulation of *Kif5b* results in defective meiotic prophase I in mouse oocytes and aberrant chromosomal segregation in mitotic cells. These results suggest that KIF5B is critical for chromosomal stability during female meiotic and mitotic cell division.

## Results

### KIF5B is critical for germinal vesicle breakdown (GVBD) and polar body exclusion

**Figure 1:**
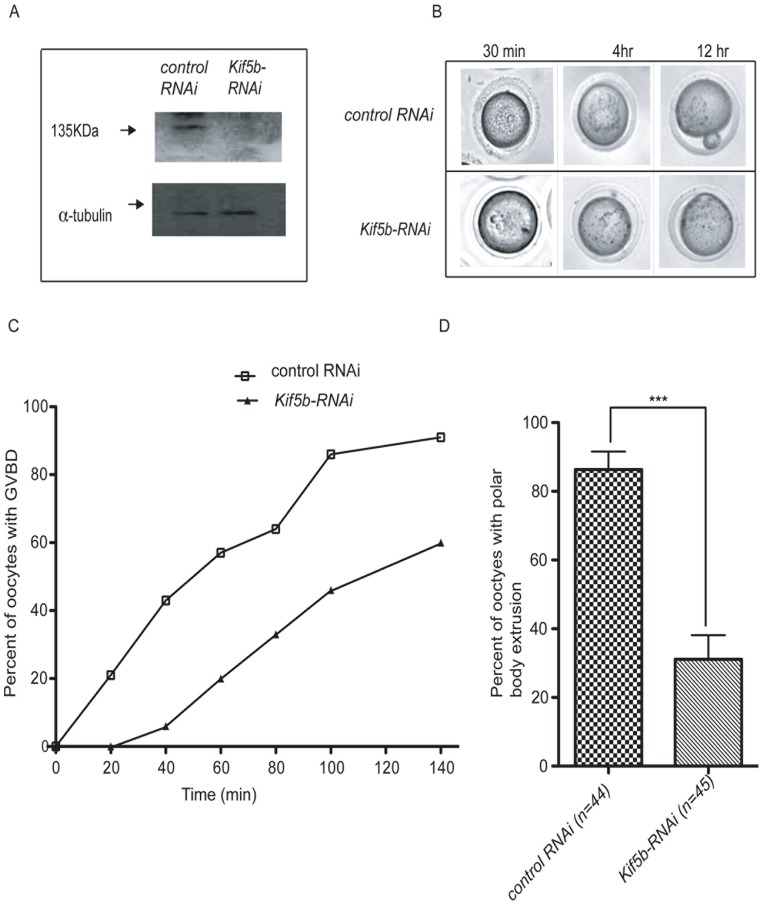
Downregulation of KIF5B blocks Metaphase I in female meiosis. A) Western blot analysis of oocytes treated with siRNA against *Kif5b* or control (luciferase) B) Live images of oocytes at different times after microinjection with *control-RNAi* and *Kif5b-RNAi*. C) Kinetics of GVBD in oocytes at different time points. D) Estimated percentage of polar body extrusion (PBE) (control (n = 44) and *Kif5b-RNAi* (n = 45).

To determine if KIF5B is critical for meiotic progression, meiotically competent germinal vesicle intact oocytes were microinjected with RNAi against *Kif5b* and monitored for 16 hr. Microinjection of the oocytes with RNAi against *Kif5b* resulted in significant downregulation of the protein, as shown in Figure 1A. We observed a substantial delay in germinal vesicle breakdown (GVBD) in 50% of the oocytes microinjected with RNAi against *Kif5b* as compared to control (Figure 1B and C). Note that this appears to be a delay rather than failure of GVBD in a large percentage of cells. *Kif5b-RNAi* injection resulted in only 31% of oocytes extruding the first polar body at 16 hr, which was significantly fewer than the 90% of wild-type oocytes extruding the first polar body at 16 hr, (P<0.01) (Figure 1B and D). Importantly, our results suggest that RNAi-mediated knockdown of *Kif5b* prevented oocytes from progressing to MII at 16 hr, indicating that KIF5B is necessary for the first meiotic division.

### KIF5B is essential for metaphase alignment and integrity of mitotic spindle

**Figure 2:**
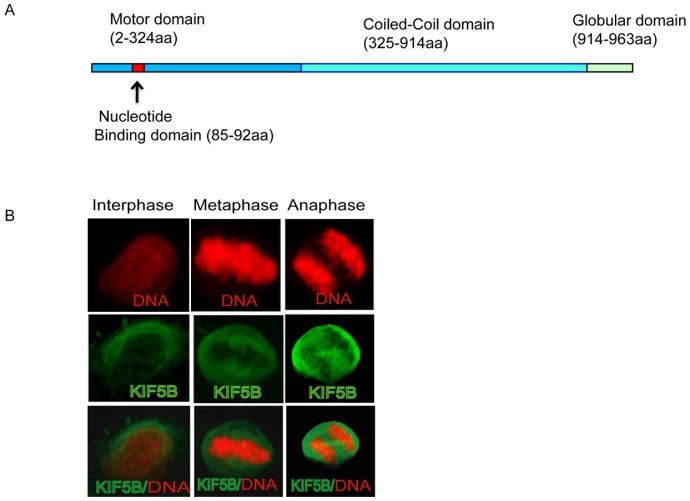
Characterization of mouse KIF5B during cell division. A) Schematic diagram of KIF5B domains based http://www.ensembl.org sequence database. Blue, red, cyan and green boxes indicate motor, nucleotide binding, coiled-coil and globular domains respectively. B) Dynamic localization of KIF5B during different stages of cell division. Note that red and green represent DNA and KIF5B respectively.

Given the suggested role of KIF5B in meiotic cell division in oocytes, we characterized the role of this protein in mitosis. Mitotic chromosomal segregation is orchestrated by the dynamic interaction between spindle microtubules and the kinetochore. KIF5B motors may crosslink adjacent spindle microtubules and slide across them, but it is unknown whether KIF5B or other motors contribute to establishing these microtubule polarity patterns to maintain proper metaphase alignment. First, we perused the protein sequence of *Kif5b* (http://www.ensembl.org), and noted that the amino-terminal domain of *Kif5b* comprises a motor head domain (2 to 324 amino acids (aa) with a nucleotide-binding domain (85–92aa) and microtubule-binding activities; whereas the carboxy-terminal end comprises coiled-coil (325–914aa) and globular domains (914–963aa) as shown Figure 2A. Furthermore, our initial characterization of KIF5B shows that KIF5B was localized in the cytoplasmic compartment of the cells during mitotic cell division (Figure 2B).

**Figure 3:**
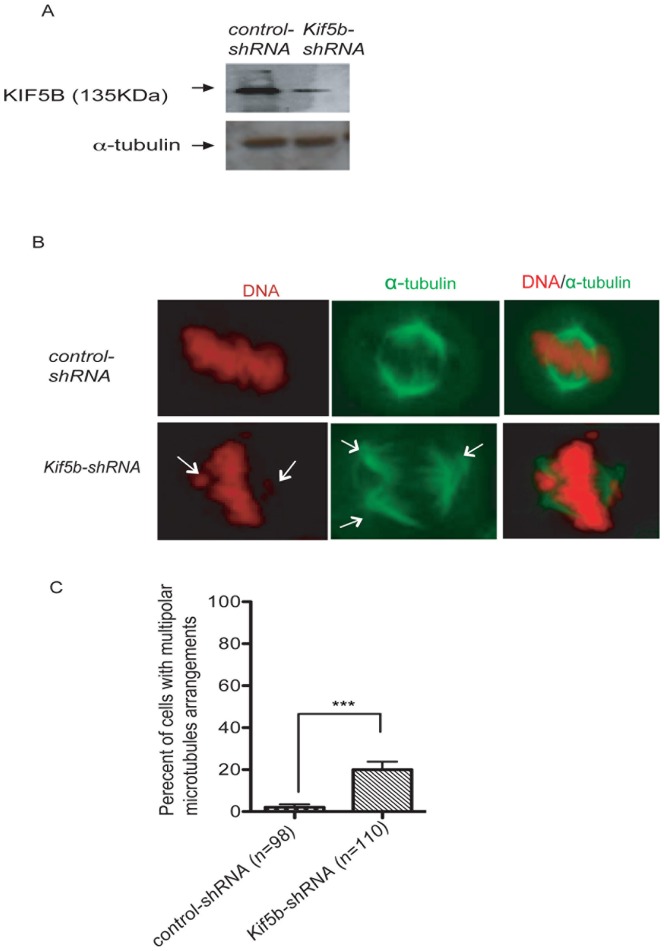
Downregulation of KIF5B leads to chromosome misalignment. A) Western blot showing the efficiency of *Kif5b-shRNA-*mediated knockdown with whole- cell extracts prepared from HeLa and control cells transfected with *control-shRNA*. B) Cell division-dependent localization of microtubules (green) and DNA arrangement (red) in *Kif5b-shRNA* versus control. Note that the arrow indicates examples of tripolar arrangement in microtubules. C) A statistically significant difference was observed for the estimated percentage of cells with multipolar arrangements of microtubules in *Kif5b-shRNA* knockdown cells (n = 110) versus wild type (n = 98)(*** p<0.0001). The Graph Pad Prism program was used for data analysis employing Mann Whitney statistical methods.

**Figure 4:**
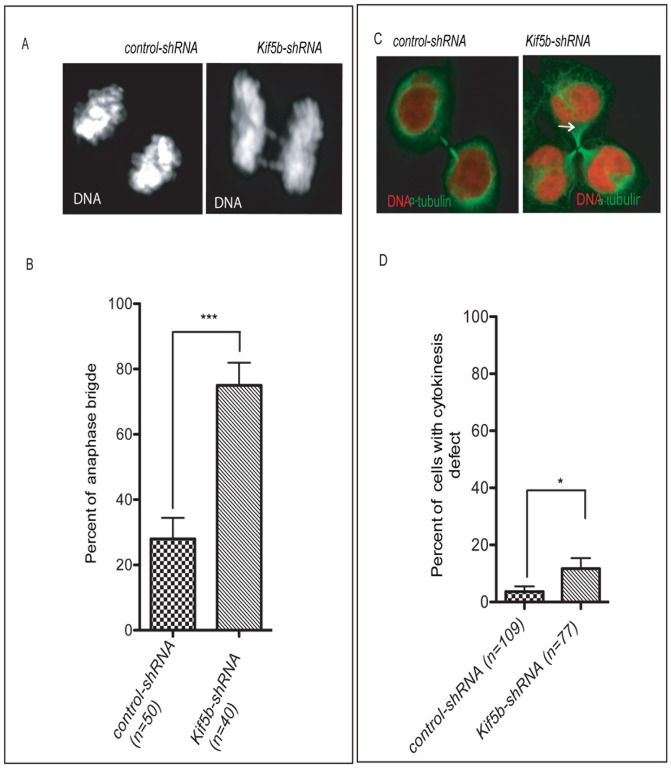
KIF5B is required for chromosome segregation and cytokinesis. A) Examples of control cells with normal anaphase chromosome separation (n = 50). B) Anaphase bridges shown with arrow in *Kif5b* downregulated cells (n = 40). Note that *** represents P<0.0001 statistically significant differences between control cells that are transfected with scrambled shRNA and *Kif5b* knockdown cells in chromosome arrangements at anaphase stage. C) Representative images of cells with normal and abnormal cytokinesis. D) The percentage of cells with cytokinesis failure is significantly increased (* P<0.01) in *Kif5b-shRNA-*mediated knockdown cells (n = 77) compared to control cells transfected with scrambled shRNA cells (n = 109). Note that the white arrow points to an example of aberrant cytokinesis, red represents DNA, and green is α- tubulin.

To assess the role of KIF5B in metaphase alignment and microtubule arrangement, we employed 5′UTR-shRNA-mediated knockdown of *Kif5b*. The results show that the expression level of KIF5B protein in HeLa cells is reduced by 81% (estimated using Image J) after *Kif5b*-shRNA transfection (Figure 3A). *Kif5b* knockdown in HeLa cells (n = 110) results in significantly more multipolar arrangements of microtubules (P<0.0001) when compared to control cells (n = 98), as shown in Figure 3B and C. *Kif5b* knockdown also results in a significant increase in lagging chromosomes during anaphase (p<0.001) (Figure 4A, B). Cell division may be inhibited by the presence of chromatin in the cleavage site, which would result in cytokinesis failure and the formation of bi- and multi-nucleated cells [18], [19]. We therefore asked if cells depleted of *Kif5b* were more prone to cytokinesis failure. We determined that *Kif5b* knockdown cells (n = 77) have a significant increase in cytokinesis failure compared to control cells (n = 109) (P<0.01) (Figure 4C, D).

### KIF5B suppresses mitotic centrosome amplification

**Figure 5:**
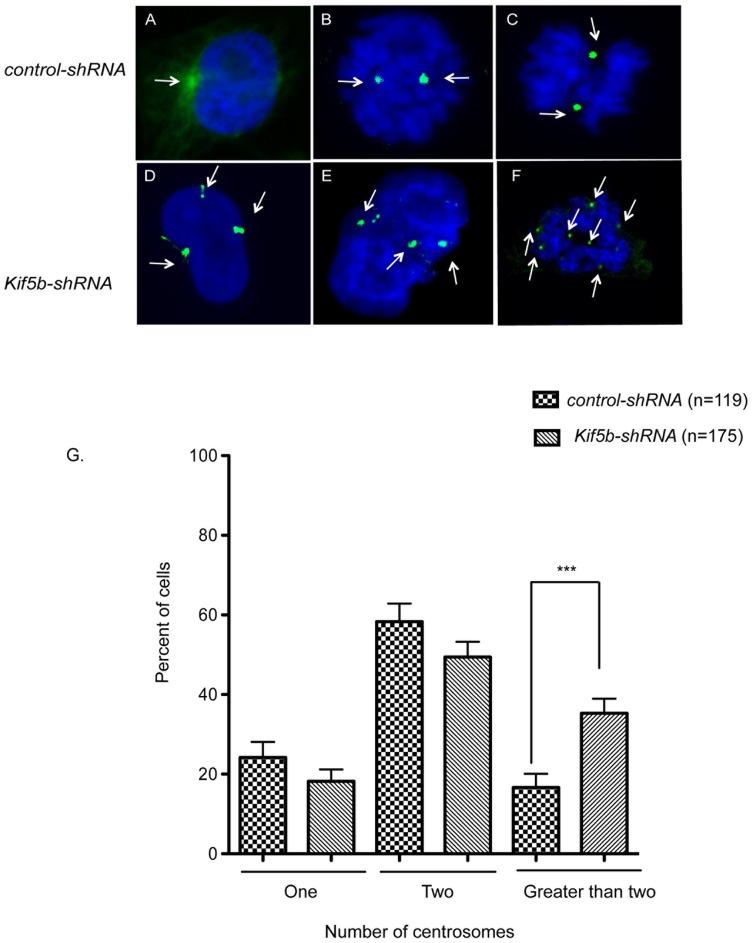
KIF5B suppresses centrosome amplification. A–C) Control cells during different stages of cell division with one and two centrosomes. D–F) Representative images of cells with *Kif5b-shRNA-*mediated knockdown with more than two centrosomes. The DNA is stained with DAPI (blue), and centrosomes are stained with γ-tubulin (green). The arrow indicates the centrosome in each cell. G) The representative number of cells analyzed for centrosomes in control cells (n = 119) and *Kif5b-shRNA* knockdown cells (n = 175). Cells with greater than two centrosomes were significantly enriched by *Kif5b-shRNA-*mediated knockdown (*** P<0.0001). The Graph Pad Prism program was used for analysis of the data and employed the Mann Whitney test.

In early mitosis, microtubule-dependent forces are applied to the centrosome by motor proteins that organize and form the bipolar spindle [20]. The centrosome plays a vital role in mitotic fidelity, ensuring establishment of bipolar spindles and balanced chromosome segregation. In order to assess the effect of *Kif5b* downregulation on centrosome integrity, we stained control cells (HeLa) and *Kif5b*-depleted cells with antiserum raised against γ-tubulin (Figure 5A). We found similar numbers of cells with one or two centrosomes per cell in *Kif5b* (n = 175) knockdowns and controls (n = 119). In contrast, the numbers of cells with more than two centrosomes was significantly increased in *Kif5b* knockdown cells compared to wild-type controls (P<0.0001) (Figure 5G).

## Materials and Methods

### Mouse stimulation, oocyte collection, culture and microinjection

Ovaries were collected from 8-week-old B6SJLF1/J (The Jackson Laboratory #100012) female mice 44–48 hr after priming with 5 IU pregnant mare serum gonadotropin. To isolate oocytes, ovaries were punctured with 27-gauge needles in M2 medium. The cumulus cells were removed by treating with hyalurionidase, and germinal vesicle (GV) intact oocytes were collected and cultured further in M2 medium supplemented with 50 μg/ml dibutiryl cyclic AMP (dbcAMP; Sigma) which prevents the resumption of meiotic maturation.

### Small Interfering RNA (siRNA) for oocytes microinjection

For the siRNA studies, the 21-mers of the siRNA duplexes directed against *Kif5b* with reference sequence NM-008448 were used to select siGenome ON-TARGETplus SMART pool (catalog number L-040710-01-0005) and were synthesized by Dharmacon Research Inc. (Lafayette, CO). All siRNA were resuspended in RNase – free water for the final concentration of 20 μM, and the siRNA was aliquoted into small volumes and stored at −80°C until the experiment was performed. All RNAi sequences are listed in Table 1.

**Table 1:** Sequences of RNAi and shRNA used for this study to knock down *Kif5b* and control.

Name	Type	Sequence (5′-3′)
*Kif5b-RNAi*	Sense	gagcuaaaccguuggcguauu
		gcaagaaguagaccggauauu
		caacagacaugucgcaguuuu
		caggacagaugaaguauaauu
	Antisense	uacgccaacgguuuagcucuu
		uauccggucuacuucuugcuu
		aacugcgacaugucuguuguu
		uuauacuucaucuguccuguu
*control-RNAi*	Sense	gauuauguccgguuauguauu
	Antisense	uacauaaccggacauaaucuu
*Kif5b-shRNA*	Sense	caccggatcggaagtgagcattagcgaacctaatgctcacttccgatcc
	Antisense	ggatcggaagtgagcattaggttcgcctaatgctcacttccgatcc
*control-shRNA*	Sense	ggcatattccctcgggcaactcgcgatccaagcgaaatccggtatacta
	Antisense	gtcgcgcacggtctgccggatcgatagaatcttaacgctgtcatat

### Microinjection

Germinal vesicle-stage oocytes were microinjected in droplets of pre-warmed (37°C) dbcAMP-supplemented M2 medium under mineral oil. Microinjections were carried out on a Leitz Labovert FS (Ernst Leitz Wetzlar GMBH, Germany) inverted microscope. GV-stage oocytes were immobilized using a holding pipette, and the tip of the injection pipette was introduced across the zona pellucida. About 10pl of pure RNAi solution was injected per oocyte. Injected oocytes were kept in M2 medium (Sigma) supplemented with dbcAMP. The resumption of meiotic maturation (GVBD) was triggered by releasing the oocytes into a drug-free M16 medium (Sigma Aldrich, USA) and incubated in a humidified atmosphere of 5% CO_2_ at 37°C.

### Antibodies

Primary antibodies: KIF5B, goat anti-KIF5B (1∶100 dilution; Abcam, USA); α-tubulin, mouse anti-α-tubulin (1∶100; Abcam, USA); γ-tubulin, mouse monoclonal anti-γ-tubulin (Sigma Aldrich, USA) and FITC-conjugated secondary antibodies were used from the appropriate species (Jackson ImmunoResearch Inc.) to stain the cells. All secondary antibodies were used at a 1∶100 dilution.

### Preparation of shRNA

Online tools from Invitrogen were used to design an shRNAmir to target the 5′UTR region of *Kif5b.* Sense and antisense oligonucleotides were annealed together (95°C, 30”; 72°C, 2′; 37°C, 2′; 25°C, 2′) and cloned into the *Bam*HI and *Eco*RI sites of pSIREN-retroQ (gift from Dr. Daniel DiMaio, Yale University). All shRNA sequences are listed in Table 1.

### Cell Culture and Transfections

HeLa cells were routinely maintained in DMEM plus 10% FBS supplemented with 100U/ml penicillin and streptomycin at 37°C and 5%CO_2_ in a water-jacketed incubator. Transfections were performed by the calcium chloride procedure (Invitrogen, Carlsbad, CA). For establishing shRNA, HEK293T cells grown in DMEM plus 10% FBS medium supplemented with 4 mM L-glutamine, 20 mM HEPES, pH 7.3 and 2×10^5^ cells were transfected with 4 µg/ml of each construct and 8 µg/ml pVpack-VSVG, 6 µg/ml pVpack-GP (Stratagene) and supernatant was harvested from infected cells at days 2, 3, 4, and 5 post-infection. For preparation of virus particles, cell culture supernatants were first pre-cleared from cell debris using a 0.45 µm filter. For downregulation of *Kif5b*, 30% confluent HeLa cells were infected with the retrovirus for 3 hr, the medium was changed to select for puromycin resistance using 0.8 µg/ml, and the cells were subcultured for the next 48 hr.

## Discussion

### The Meiotic Role of KIF5B

Mammalian oocytes are notorious for high rates of chromosomal abnormalities [21], resulting in subsequent embryonic aneuploidy, infertility, and congenital defects. Unfortunately, the necessary components for and regulation of successful oocyte maturation remain unknown. Previous findings showed that targeted disruption of mouse *Kif5b* impaired embryonic development [17], [22]. In this work, we have shown that downregulation of *Kif5b* from germinal vesicle- stage oocytes delays GVBD. As a result, extrusion of the first polar body is strongly inhibited. Our observation is further supported by previous findings demonstrating that spindle defects lead to the inhibition of polar body extrusion in meiosis [23]. This phenotype indicates that *Kif5b* is clearly necessary for the progression of meiosis I in oocytes.

Apart from its possible role as a molecular motor, our observations suggest that KIF5B might also have an additional, and possibly complementary, function as a critical component in kinetochore assembly and the promotion of oocyte cell development.

### The Mitotic Role of KIF5B

Genomic stability requires faithful chromosome segregation during mitosis and is ensured by two cooperating mechanisms. First, centromere geometry is inherently biased toward bi-oriented attachment of chromosomes to spindle microtubules, thereby constituting a prevention mechanism against mal-orientation [24], [25]. Second, the role of KIF5B as a molecular motor is suggested by its close homology with the other kinesin 1 family members [26]. In this function, it may contribute to generating the force that is required for spindle bipolarity and chromosome alignment at metaphase [27], [28], [29]. This interpretation is consistent with our observations because in the majority of *Kif5b* downregulated mitotic cells, centrosomes were amplified by failure of cytokinesis as a major source of lagging chromosomes. Furthermore, KIF5B localized in the cytoplasm and associated with microtubules following the spindle bipolarity fashion, similar to its Drosophila homologue KLP3A in cytokinesis during Drosophila meiosis [30], [31]. This pattern of cellular localization has also been observed with KIF14 [32] but not with KIF4A, which is predominantly localized in the nuclear matrix and is associated with chromosomes during mitosis [33].

### KIF5B is required for Centrosome Integrity and Spindle Bipolarity

Elevated frequencies of chromosome mis-segregation are a direct indicator of chromosomal instability [34], [35]. We showed that *Kif5b* silencing disrupts proper chromosomal segregation, which is similar to the phenotype of KIF14 [32]. The establishment of a bipolar spindle and dynamic interactions with kinetochores are important for proper mitotic chromosome segregation [36], [37] **.** There are two possible mechanisms by which centrosome abnormality promotes aneuploidy and malignant transformation. We observed that cells with multiple centrosomes are likely to adopt multipolar spindles that result in unequal distribution of the chromosomes to the daughter cells. As a result, the centrosome amplification may enhance chromosome instability (Figure 5A) and eventually contribute to formation of lagging chromosomes at anaphase (Figure 4A). The other possible mechanism by which lagging chromosomes can appear during anaphase is if a single chromatid establishes a connection to both spindle poles. This state has been explored previously by producing mono-centromeres either by separating sister chromatids [38] or by preventing genome replication before mitosis [39]. Such centromeres can congress to the metaphase plate but do not segregate well during anaphase because the single centromere has established connections with both spindle poles. In addition to cytokinesis failure, we have also observed an increase in cell death in response to moderate *Kif5b* silencing (data not shown). One possibility is that KIF5B may indirectly activate spindle assembly checkpoint (SAC) that is required for the regulation of mitotic cell cycle progression to ensure mitotic fidelity and to detect mitotic spindle abnormalities. Such a spindle assembly checkpoint could operate throughout mitosis and would be diminished or eliminated as a consequence of *Kif5b* silencing. Our observations in *Kif5b*-downregulated cells are consistent with the possibility that KIF5B could facilitate the proper positioning of the centromere by using an unidentified adaptor protein to move the chromosomes along the microtubules, which is also in line with other findings indicating that mitotic kinesins cooperate to drive sister chromatid separation during anaphase [40].

In total, we propose that KIF5B may function as a molecular motor for the movement of the kinetochore complex using currently unidentified adaptor proteins and/or DNA to interact with other accessory proteins that contribute to higher-order organization of metaphase chromosomes. Its depletion might thus be expected to result in a lagging chromosome, giving rise to the observed chromosome segregation defects and centrosome amplification. Consistent with such a role of KIF5B, we find multiple defects both in chromosome structure and mitotic spindle organization.
